# Cefepime-Induced Delirium

**DOI:** 10.7759/cureus.15505

**Published:** 2021-06-07

**Authors:** Francisco J Somoza-Cano, Abdul Rahman Al Armashi, Anastasiia Weiland, Deema Chakhachiro, Keyvan Ravakhah

**Affiliations:** 1 Internal Medicine, St. Vincent Charity Medical Center, Cleveland, USA

**Keywords:** cefepime, pressure ulcers, infectious disease medicine, general medicine pharmacology, cefepime-induced neurotoxicity

## Abstract

Cefepime is a fourth-generation cephalosporin usually reserved to treat severe infections or those caused by multi-resistant microorganisms. Neurotoxicity is attributed to its ability to cross the blood-brain barrier and produce gamma-aminobutyric acid antagonism. Neurological symptoms may range from mild somnolence to seizures and coma. Our patient is an 88-year-old man who presented from a nursing home due to worsening pressure ulcers. After cefepime was started, he started developing worsening altered mental status and hallucinations. Cefepime was discontinued and his neurological symptoms improved shortly afterward. He was discharged to a long-term acute facility for antibiotic therapy where he recovered. Our case illustrates a commonly missed side effect of cefepime. Prompt recognition of this adverse effect is paramount to prevent disease progression and avoid permanent neurological damage.

## Introduction

Cefepime is a broad-spectrum, fourth-generation cephalosporin that binds to and inactivates penicillin-binding proteins located on the bacterial cell wall [1]. It is known to cause central nervous system depression by its ability to cross the blood-brain barrier (BBB) and antagonize gamma-aminobutyric acid class A (GABA-A) in a concentration-dependent manner. This may be further worsened by impaired kidney function and concomitant diseases (e.g., sepsis, chronic inflammation), inhibiting drug metabolism and leading to increased serum concentration [2]. The incidence of neurotoxicity ranges from 1% to 15% due to the underrecognition of this phenomenon by clinicians [3].

## Case presentation

An 88-year-old man with a past medical history of hypertension, hyperlipidemia, and peripheral artery disease presented from a nursing home after a one-week history of worsening fever and right buttock pain. On presentation, his vital signs were stable and he was alert and oriented to all spheres. Significant physical examination findings included a stage 4 pressure ulcer on the right buttock and stage 2 pressure ulcers on bilateral heels with foul smell and purulent discharge. Initial workup revealed a total leucocyte count of 4.5 K/µL (reference range: 3.9-11 K/µL) with 11% bands (reference range: 0-5%), toxic granulation, and elevated metamyelocytes at 1% (reference: 0%). Creatinine was 2.7 mg/dL (reference range: 0.3-1.5 mg/dL), increased from a baseline of 1 mg/dL. Blood urea nitrogen (BUN) 45 mg/dL (reference range: 5-24 mg/dL) and electrolytes were unremarkable. Lactic acid was 0.7 mmol/L (reference range: 0.8-2.0 mmol/L) and inflammatory markers showed a C-reactive protein of 70.1 mg/L (reference range: 0-3 mg/L), erythrocyte sedimentation rate of 55 mm/hour (reference range 0-20 mm/hour) and a procalcitonin of 0.05 ng/mL (reference range: less than 0.05 ng/mL). Cefepime and vancomycin were empirically started after dose adjustment for kidney function and General Surgery debrided his pressure ulcers. Wound cultures grew *Proteus mirabilis*,* Pseudomonas aeruginosa*,* Escherichia coli*,and *Corynebacterium aurimucosum*. All were sensitive to cefepime, so the antibiotic was continued and vancomycin was halted. Inflammatory markers and bandemia were downtrending, but on day 10 of admission, he became unresponsive. His vital signs were within normal limits. After a sternal rub, the patient became verbal but incoherent. He reported complex visual hallucinations and a conversation with his deceased wife. He had no focal neurological deficits but was oriented only to person. Brain CT and MRI showed no acute intracranial processes. Repeated laboratory workup revealed normal levels of electrolytes, blood glucose, thyroid-stimulating hormone, and ammonia. Creatinine and BUN had mildly improved to 2.6 mg/dL and 40 mg/dL, respectively. After medication review, it was noted he was on pregabalin for chronic pain. This drug was discontinued but his mental status did not improve. Given the ongoing acute kidney injury (AKI) and the antibiotic of choice, cefepime neurotoxicity was suspected. The Naranjo adverse drug reaction probability scale was 8 points, making it probable [4]. Cefepime was switched to meropenem and the patient’s mentation returned to baseline within 48 hours. He was discharged to a skilled nursing facility where he completed a long-term antibiotic course.

## Discussion

The neurotoxic effects of cefepime have been described since its release to the market in the 1990s. Under physiological conditions, about 10% of the serum’s cefepime crosses the BBB. However, decreased protein binding, increased organic acid accumulation, renal impairment, and systemic inflammation can increase this transfer up to 45% [2]. The main risk factor for neurotoxicity is AKI, especially when the dosing is not adequately adjusted. Renal excretion of cefepime is up to 85%, while only a small portion of it is hepatically cleared. Nevertheless, up to 26% of patients may present with neurotoxicity even with the appropriate dosing, as seen in our patient [3,5-8]. In our patient, cefepime toxicity was not the only potential offending agent. Hospital delirium due to advanced age, sepsis, uremia, and the concomitant use of pregabalin could all cause an acute mental status change in our case.

The clinical manifestations of this phenomenon may range from mild delirium to seizures and coma. The most important intervention is cefepime discontinuation, but dose reduction, treatment with antiepileptic drugs, benzodiazepines, or hemodialysis have been proven as adequate strategies if the antibiotic must be continued. Clinical improvement usually occurs after two days of discontinuation. However, this may be skewed if the kidney dysfunction is severe enough or the integrity of the BBB has been compromised. Advanced age, presence of chronic diseases, and ongoing systemic inflammation may cause this damage to the BBB. Permanent neurological deficits are rare but have been documented [2,3].

Finally, the Naranjo algorithm, or Adverse Drug Reaction Probability Scale, is a proven method to assess a causal relationship between a clinical event and a drug [4]. Our patient had a probable score of 8 points. Additionally, Bradford Hill criteria for causation is another tool that can be useful in establishing evidence of a causal relationship between a cause and an observed effect [9]. Hill’s criteria applied to our case support the connection between cefepime and neurotoxicity. Proven evidence of cefepime’s neurotoxic effect, a temporal relationship between initiation of therapy and symptom onset, worsening course with medication continuation, and evidence of reversibility in a variety of different patient populations support the aforementioned causality [2,8].

## Conclusions

This case illustrates cefepime’s potential for inducing an acute mental status change and the associated risk factors. Physicians should draw attention to findings of neurotoxicity during cefepime therapy and not get misled with other acute disease processes as permanent neurological damage may occur if discontinuation is delayed.
